# Case Report: Limbic encephalitis following treatment with durvalumab for small-cell lung cancer

**DOI:** 10.3389/fimmu.2023.1278761

**Published:** 2023-10-16

**Authors:** Thomas T. Moss, Knut Stavem, Astrid Aandahl, Anne S. Gløersen, Bjørn H. Grønberg, Kirill Neumann, Christian A. Vedeler, Christofer Lundqvist

**Affiliations:** ^1^ Department of Neurology, Akershus University Hospital, Lørenskog, Norway; ^2^ Pulmonary Department, Akershus University Hospital, Lørenskog, Norway; ^3^ Health Services Research Unit, Akershus University Hospital, Lørenskog, Norway; ^4^ Institute of Clinical Medicine, University of Oslo, Oslo, Norway; ^5^ Department of Immunology and Transfusion Medicine, Akershus University Hospital, Lørenskog, Norway; ^6^ Department of Clinical and Molecular Medicine, Norwegian University of Science and Technology (NTNU), Trondheim, Norway; ^7^ Department of Oncology, St. Olavs Hospital, Trondheim, Norway; ^8^ Department of Neurology, Haukeland University Hospital, Bergen, Norway; ^9^ Department of Clinical Medicine, University of Bergen, Bergen, Norway

**Keywords:** durvalumab, small-cell lung cancer, Encephalitis, diabetes, adverse effects, Immunotherapy

## Abstract

**Background:**

Durvalumab is an immune checkpoint Inhibitor (ICIs) that is used in the treatment of malignant tumors, such as lung cancer and melanoma. ICIs are associated with immune-related adverse events including autoimmune encephalitis, although both paraneoplastic phenomena and ICI treatment may lead to autoimmunity.

**Case presentation:**

We describe a 72-year old male patient with small-cell lung cancer, who during adjuvant treatment with Durvalumab developed GABA_B_R_1_ and GAD65 antibodies and both diabetes and autoimmune limbic encephalitis. Because he was followed prospectively as part of a treatment study, we had access to repeated serum samples and cognitive assessments over time prior to developing encephalitis and diabetes, in addition to later assessments. A high titer of GABA_B_R_1_ antibodies appeared early, while GAD65 antibodies appeared later with a lower titer in parallel with the development of diabetes. As he subsequently developed clinical signs of encephalitis, verified by EEG and brain MRI, he also had CSF GABA_B_R_1_ antibodies. Durvalumab was discontinued and steroid treatment with subsequent plasmapheresis were started, resulting in reduction of both CSF and serum antibody levels. Clinical signs of encephalitis gradually improved.

**Conclusion:**

This case illustrates the importance of being aware of possible serious autoimmune adverse reactions, including neurological syndromes such as encephalitis, when treating patients with high risk of para-neoplasia with ICIs. In addition, the case shows the development of autoantibodies over time.

## 1 Introduction

Treatments with immune checkpoint inhibitors (ICIs) in monotherapy or combined with chemo or radiotherapy (1), in particular in lung cancer or malignant melanoma, have been followed by reports of adverse reactions, including immune-related adverse events (irAEs) (2), or neurological immune-related adverse events (n-irAEs) (3). Severe n-irAEs may occur with ICIs and can be focal encephalitis or meningoencephalitis with variable outcomes, from full recovery to death (4). Early symptoms can be non-specific, and therefore timely diagnosis may be a challenge.

Durvalumab is an ICI that targets the programmed cell death ligand 1 (PD-L1) pathway, and in combination with etoposide and cisplatin or carboplatin, is established as first-line therapy for patients with extensive small cell lung cancer (SCLC) (5).

Recent case reports have reported durvalumab-associated encephalitis during treatment for SCLC (6–8), leading to increased attention to symptoms of disorientation, cognitive impairment, or seizures in SCLC patients who receive immunotherapy.

We present a patient with extensive SCLC, who after therapy with durvalumab presented with diabetes and then limbic encephalitis. Because he participated in a clinical trial, serial cognitive tests and serum samples collected at several time points prior to the development of encephalitis were available for analysis.

## 2 Case description

The patient was a 72-year old man, who until recently had been working full time in a cognitively demanding job. In 2009, he had an anterior-wall myocardial infarction and emergency PCI. He had surgery for hernia in the spring of 2022 but was otherwise healthy. He had smoked for 35 years, until 2007.

### 2.1 Lung cancer and treatment

**Figure 1:**
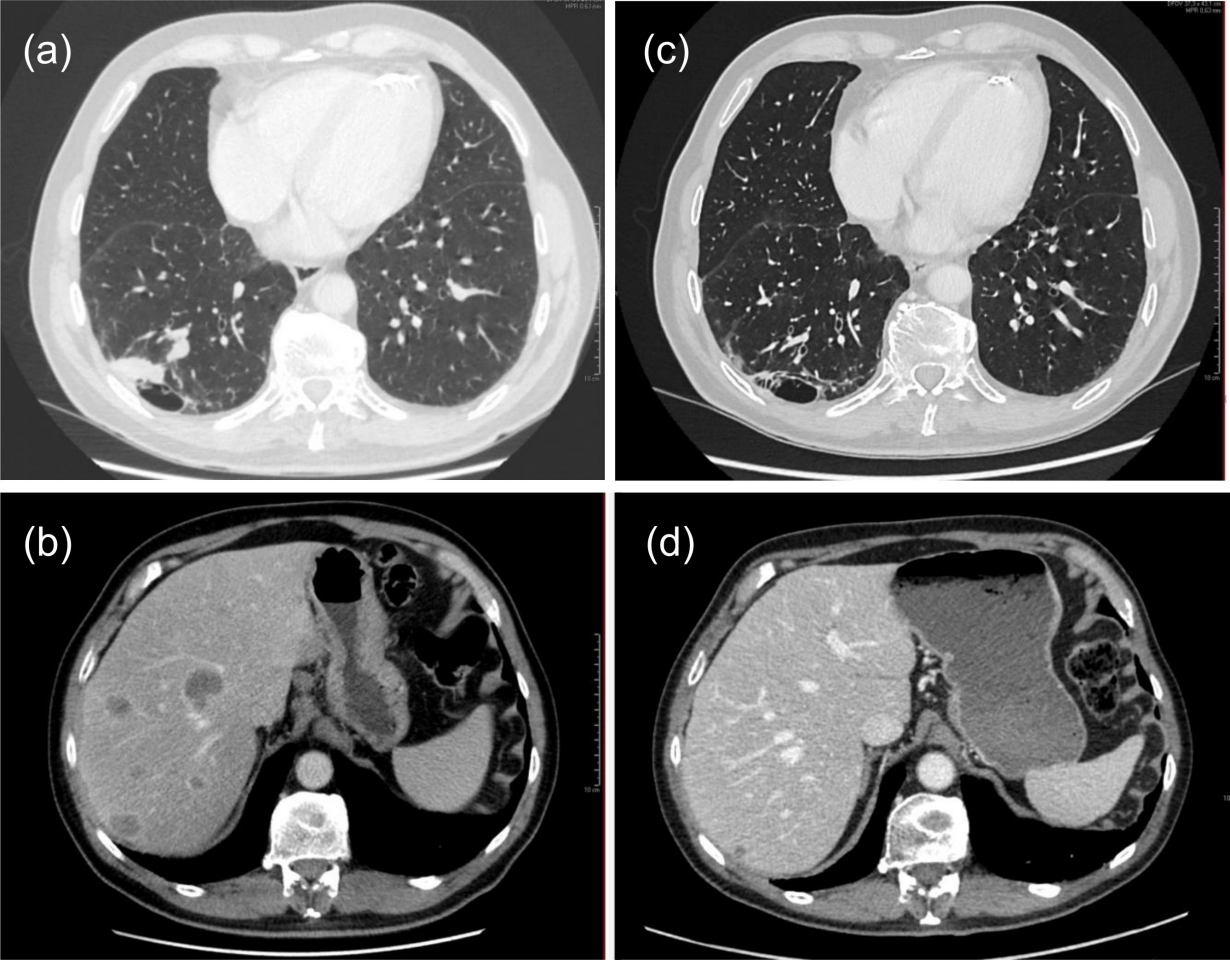
CT thorax and abdomen at diagnosis: **(A)** tumor dorsolateral in the right lower lobe and **(B)** multiple lesions in the liver at diagnosis. After four cycles of combined chemotherapy/immunotherapy showing response to therapy: **(C)** Reduction in size of nodule in right lower lobe to 4 mm, and **(D)** considerable reduction in size of liver lesions compared to before therapy.

In May 2022 (**Table 1**; week 0), 2 months after hernia surgery, he had pain in the left flank and groin, which disappeared after a few hours, and tenderness in the left axilla/hemi-thorax. CT thorax showed four potentially malignant lesions dorsolaterally in the right lower lobe, with signs of infiltration of the thoracic wall and enlarged mediastinal lymph nodes. The largest pulmonary lesions were 3x1.5 cm and 2x2 cm, respectively (**Figure 1A**). CT abdomen showed multiple liver metastases; the largest with a diameter of 5.5 cm (**Figure 1B**). After ultrasound-guided liver biopsy, he was diagnosed with extensive stage small cell lung cancer (ES-SCLC). He was in good general condition, Eastern Cooperative Oncology Group (ECOG) performance status 0.

**Table 1:** Changes over time of treatment of the patients’ pulmonary disease, cognitive function, antibody titers, diabetes (HbA1c) and encephalitis treatment with steroids and plasma exchange.

Weeks after diagnosis of lung cancer	0	4	6	8	12	16	21	25	29	33	37	39	43	51
Treatment for lung cancer, cycles														
Durvalumab	1	1		1	1	1	1	1	1					
Etoposid/Carboplatin	1	1		1	1									
Treatment response (RECIST)			Partial response			Partial response		Partial response		Stable disease			Stable disease	Progressive disease
ECOG score	0	0		1-2^*^	1	0	0	1	1	1				
S-NSE (µg/L, reference <16)	48							9.9						
Encephalitis symptoms										(+)**^§^**	+	+++	++	+
Cognitive score														
MOCA (0–30)	29					24		27					19	22
MMSE (0-30)										26			15	
Antibodies, titer														
GABA_BR_1/2 serum	1/1000	1/1000				1/10	1/10			1/10		1/10	Negative	
GABA_BR_1/2 CSF												1/10	1/1	
GAD65 serum	Negative	Negative				1/10	1/10			1/10		1/10	Negative	
GAD65 CSF												Negative	Negative	
HbA1c (mmol/mol)	41	46				64				66			72	
Treatment														
Steroids												+	+	+
Plasma exchange, sessions													5	5

RECIST, Response Evaluation Criteria in Solid Tumours; ECOG, Eastern cooperative oncology group; NSE, neuron-specific enolase; MOCA, Montreal cognitive assessment; MMSE, mini mental status examination; GABA_BR_1/2, γ-aminobutyric acid type B receptors 1-2; GAD65, glutamic acid decarboxylase 65-kilodalton isoform. (^*^COVID-19 infection, ^§^fluctuating dysphasia)

He was included in an RCT of combination therapy with carboplatin (AUC 5 mg*min/ml), etoposide 100 mg/m^2^ body surface area (BSA), durvalumab 1500 mg iv. on day 1, and etoposide 200 mg/m^2^ BSA po. on days 2–4 with or without thoracic radiotherapy and was randomized to therapy without concurrent radiation. At the start of each cycle, he had dexamethasone 8 mg for 3 days.

Shortly after the 1^st^ cycle, he developed petechia on his chest and thrombocytopenia. For the 2^nd^ cycle, he therefore received a reduced dose of carboplatin/etoposide (60% of AUC 5).

He completed four cycles of systemic therapy. CT thorax/abdomen after the 2^nd^ cycle, showed considerable reduction of lesions in the right lower lobe and lymph nodes in the mediastinum and right hilum, but new lesions in corpus of vertebra T8 and possibly a pathological fracture of the 8^th^ rib. The liver metastases were reduced in size, from 55 to 25 mm largest diameter for the largest lesion. After the 4^th^ cycle, the pulmonary lesions were smaller, with a remaining 4 mm nodule in the right lower lobe (**Figure 1C**) and reduced size of the liver metastases (**Figure 1D**), and serum neuron-specific enolase (NSE) was normalized. Brain CT did not detect brain metastases. He then continued with durvalumab 1500 mg iv. monotherapy every 4^th^ week and received the 5^th^ cycle in September 2022 (week 16). In October 2022 he received prophylactic cranial irradiation (PCI) of 25 Gy in 10 fractions and continued with durvalumab monotherapy (1500 mg iv. every 4 weeks). In December 2022, he was in good general condition, ECOG 1, but had challenges with diabetes control. CT evaluation early January 2023 showed stable disease in the thorax/abdomen, and there were no metastases on brain CT.

### 2.2 Onset of diabetes mellitus

One week later (week 17), at consultation for evaluation for PCI, he was diagnosed with diabetes mellitus with blood glucose 26.7 mmol/L, HbA1c 64 mmol/mol (reference 20–42), c-peptide 230 pmol/L (fasting reference 300-1480). A diagnosis of diabetic ketoacidosis was confirmed and insulin treatment was started.

Serum IA2 and GAD65 antibodies were not detected using an ELISA-based test from RSR Limited, (Cardiff, UK). In May 2022, before the start of immunotherapy, he had no confirmed diagnosis of diabetes.

### 2.3 Development of neurological symptoms and confusion

In January 2023, during 4 days of hospitalization for compression fracture of vertebra T7 after a fall, he had spontaneous alterations of cognition. The examination revealed no neurological abnormalities, and a brain CT was normal.

A few days later, he was admitted to the stroke unit of the hospital after a 10 min episode of dysphasia with spontaneous normalization. Brain CT, including angiography and perfusion images, showed no evidence of stroke. However, he had minor electroencephalogram (EEG) abnormalities with a left-sided frontotemporal dominance and reduced cognition, with 26/30 points on the mini mental status examination (MMSE). An epileptic seizure could not be excluded, but this was not treated. He was discharged to his home with an outpatient appointment for a new brain MRI and follow-up for treatment of his SCLC.

In February 2023 (week 41), he was admitted to the Department of Pulmonary medicine because of increasing confusion. His wife described seizure-suspect episodes with strange swallowing noises, no eye contact, and tremor-like convulsions of the upper extremities, followed by incoherent speech. In addition, his general condition had worsened, with increasing gait disturbances and imbalance, and visual hallucinations.

### 2.4 Diagnosis, treatment and follow-up

**Figure 2:**
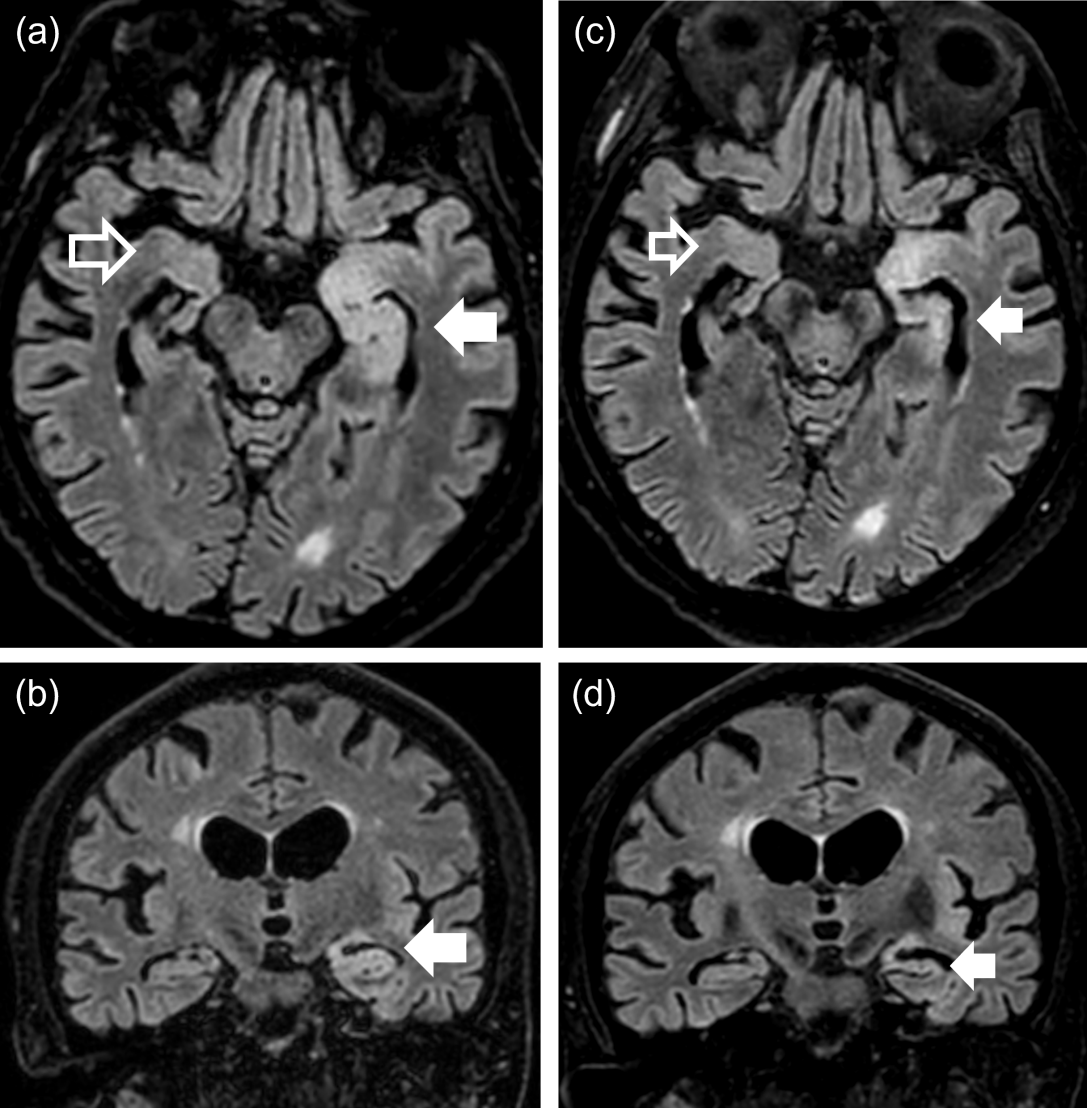
MRI images (FLAIR, 3 T) of the brain showing pre-PLEX treatment situation **(A, B)** and situation five weeks later after PLEX **(C, D)**. **(A)** Transverse section and **(B)** coronal section through temporal lobes and hippocampi showing clear hyperintense signal in temporal lobe and hippocampus on the left side (with some minor hyperintensity also on the right side). **(C)** and **(D)** show same sections after PLEX treatment with reduced hyperintensity signal in the same regions. Arrows depict hyperintense areas in left hemisphere (filled arrows) and right hemisphere (un-filled arrows).

At admission to the Department of Pulmonary medicine, he had no neurological abnormalities. During the next days, he became disoriented and periodically dysphasic. Brain MRI on day 4 after admission showed high T2/FLAIR signal and increased volume in the left temporal lobe and hippocampus with no gadolinium contrast enhancement (**Figures 2A, B**), which suggested limbic encephalitis, possibly paraneoplastic. EEG demonstrated sequences with polymorphic and semi-rhythmic theta and delta activity over both hemispheres, with a left-sided temporo-frontal maximum with sharp potentials. Lumbar puncture (day 7) with cerebrospinal fluid (CSF) analysis showed no signs of bacterial or viral encephalitis, but evidence of intrathecal IgG bands not present in serum.

He was diagnosed with autoimmune encephalitis and started treatment with intravenous methyl prednisolone on day 7, 1 g qd for 5 days and anti-seizure therapy with levetiracetam 250 mg bid and was transferred to the Department of Neurology.

Four days after starting steroid treatment, he was tired, had bilateral Babinski´s sign, but otherwise normal tendon-reflexes. He was unstable on walking, but had negative Romberg test, no ataxia of the extremities and normal motor examination. He answered questions adequately but had problems with short-term memory.

**Figure 3:**
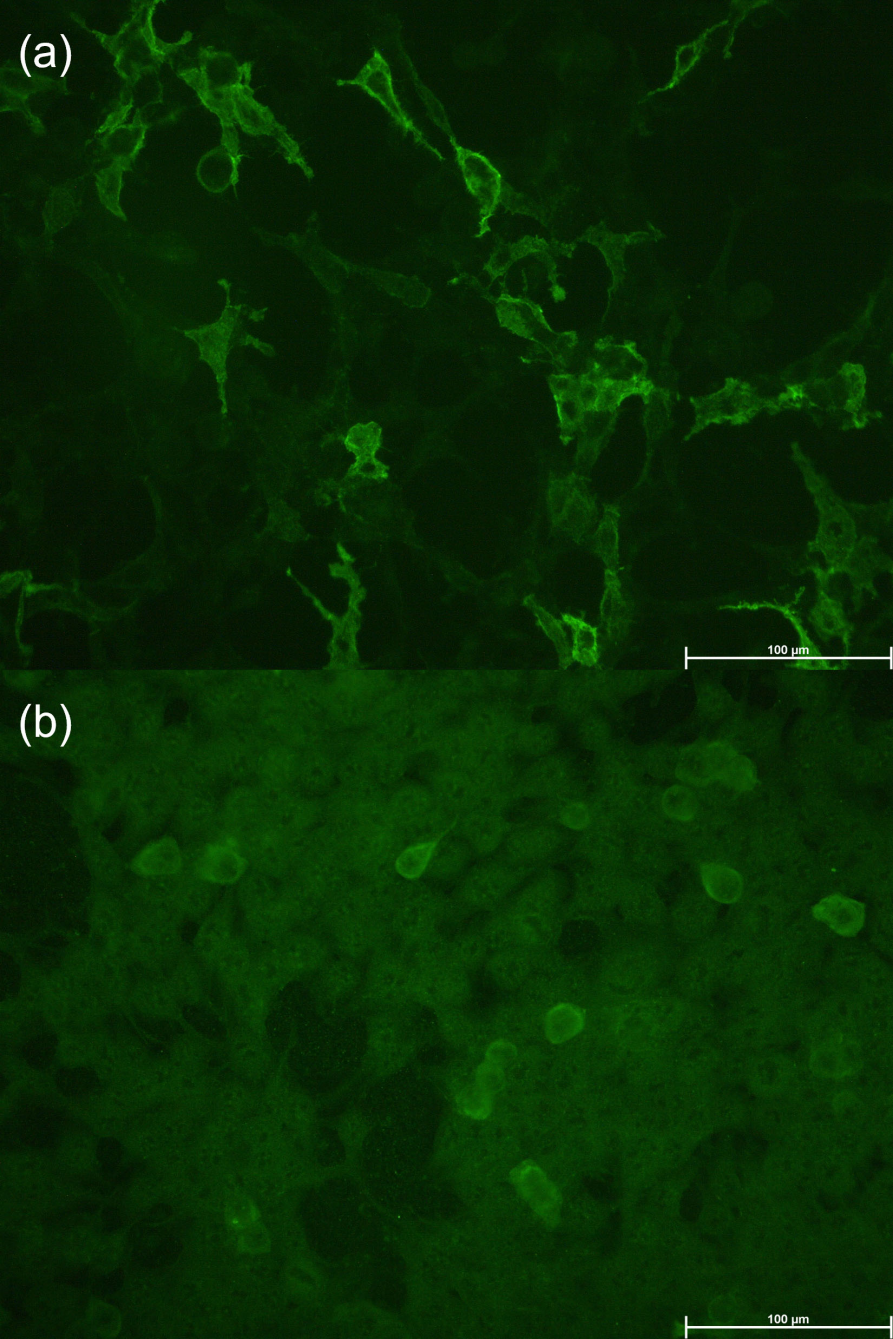
Representative images of the reaction of antibodies from the patients’ serum with cells that express GABA_B_R **(A)** and GAD65 **(B)**.

A neuro-immunological paraneoplastic and encephalitis panel analysis was positive for antibodies in serum against both γ-aminobutyric acid type β receptor 1 (GABA_B_R_1)_ and glutamic acid decarboxylase 65 (GAD65) (**Figure 3**), while CSF analysis was positive only for GABA_B_R_1_ (**Table 1**). No other encephalitis or paraneoplastic antibodies were detected using

the Euroimmun autoimmune encephalitis mosaic 6 cell-based assay (Euroimmun, Lübeck Germany) or the Ravo PNS 14 line assay (Ravo Diagnostika, Freiburg im Breisgau, Germany).

After 10 days of treatment, he still had poor balance/gait, was not oriented for time/place and person and had cognitive difficulties. There was no clinical improvement since hospital admission. Plasma exchange (PLEX) was therefore started, with sessions every other day using a Spectra Optia apheresis machine (Terumo BCT, Lakewood, CO, USA), in total five sessions. Each time, 1.2–1.3 plasma volumes were removed with 4% albumin as substitution fluid and acid citrate dextrose solution as anticoagulant. He continued with oral prednisolone 80 mg per day with gradual tapering of the dose.

After the first PLEX session, he improved cognitively, no longer had a Babinski sign, but neurological status was otherwise unchanged. Three days after final PLEX treatment, EEG revealed no focal epileptic activity, and 6 days after PLEX, brain MRI showed improvement in the left hippocampus but remaining high signal in the anterior left hippocampus and amygdala, with reduced edema (**Figures 2C, D**). Serum analysis was negative for GABA_B_R_1_ and GAD65 and CSF positive for GABA_B_R_1_, but negative for GAD65 (**Table 1**).

At hospital discharge, he was more oriented, had less gait disturbances, but still had problems with short-term memory and scored 15/30 (normal score ≥26) on the MMSE and 19/30 (normal score ≥26) on the Montreal cognitive assessment (MoCa) scale (9), suggesting moderate cognitive impairment.

He stopped with durvalumab because of what was assessed as a severe adverse immunotherapy-related event and had no further treatment for SCLC during the next months.

At ambulatory 1-year follow-up for his SCLC, he was in better general condition. Relatives reported some improvement in cognitive function, which was supported by an improved MoCa score of 22/30, indicative of mild cognitive impairment.

After hospital discharge, we had access to previous MoCa test scores and frozen serum samples from a biobank (stored at -80°C) at several time points before the diagnosis of encephalitis, collected as part of the RCT. The serum samples were screened for relevant antibodies. There was a dip in the longitudinal MoCa scores at the time of encephalitis development and a slight improvement with encephalitis treatment in parallel with the observed clinical improvement. Detectable antibody titers of GABA_B_R_1_ were found from baseline in serum and during the encephalitis phase in CSF, whereas GAD65 antibodies, using a cell-based assay with human embryonal kidney cells (Euroimmun, Lübeck, Germany), were seen only in serum from week 16 to 39 (**Table 1**). Titers were obtained by testing serial dilutions of serum (starting at 1/10), and CSF (starting at 1/1; undiluted).

## 3 Discussion

We have described a patient with SCLC, who developed an ICI treatment-associated limbic encephalitis and was treated with high dose steroids and subsequent PLEX.

Serial cognitive assessments with MoCa (9) showed a parallel reduction in cognitive function with neurological and cognitive improvement after treatment. Comparison of brain MRI before and after treatment showed a reduction of changes in the hippocampal region. Serial analyses of prospective serological samples collected as part of an RCT before encephalitis diagnosis as well as pre- and post-treatment CSF samples, demonstrated changes of GABA_B_R_1_ and GAD65 autoantibodies over time.

Anti-GABA_B_R_1_ was present with a high titer in serum before starting treatment with durvalumab, suggesting paraneoplastic association with SCLC. When the patient developed encephalitis, the serum anti-GABA_B_R_1_ titer was lower, but anti-GABA_B_R_1_ was also detected in the CSF, and it is therefore likely that this antibody was associated with development of limbic encephalitis. In a similar case report of a patient with SCLC and encephalitis after durvalumab, both serum and CSF were positive for anti-GABA_B_R antibodies (7).

GAD65 antibodies appeared late in the course of disease (16 weeks after baseline), and with a lower titer than GABA_B_R_1_. The appearance of anti-GAD65 in serum was associated with the onset of diabetes, but was not found in the CSF. When anti-GAD65 is associated with neurological disorders, such as encephalitis, high serum levels are found, whereas the more common development of diabetes is associated with lower levels that decrease with time (10). This is in line with our case where low levels of GAD65 antibodies were detected by transfected cells, and not by ELISA, and were probably associated with autoimmune diabetes and not with limbic encephalitis. Taken together, it is possible that the strong immune activation by durvalumab may first have induced autoimmune diabetes related to anti-GAD65, and secondly contributed to propagation of GABA_B_R_1_ to the CNS, which subsequently caused limbic encephalitis.

There is accumulating evidence that ionizing radiation of the brain may lead to cognitive deficits, also after low doses, possibly through mechanisms such as chronic neuroinflammation and altered immune reactions (11, 12). Radiation-induced brain injuries are often classified as acute, early-delayed or late-delayed, depending on when they occur after radiotherapy (13). Thus, PCI for SCLC may lead to late cognitive deficits (14, 15). The prevalence of such sequelae varies, because studies use different irradiation-dose and different assessment methods. Radiotherapy may also boost the effect of anti-cancer immunotherapy, both locally and systemic, although detailed mechanisms are not completely understood (16), and the clinical impact is unknown since very few patients in studies of chemoimmunotherapy in ES-SCLC have received PCI (5, 17). Our patient had brain irradiation more than 4 months after starting durvalumab treatment and the demonstration of GABA_B_R_1_ antibodies, but 4 months before onset of the symptoms of encephalitis. Therefore, we cannot exclude that the PCI may play a role in the pathogenesis here, e.g., by direct radiation-induced tissue damage, direct immuno-modulating effects, potentiation of side-effects from durvalumab.

Our patient received high-dose steroids with little or no early clinical improvement and worsening of his diabetes. For irAEs that are not corticoid-responsive, PLEX or IVIG are alternative treatments as has also previously been suggested for anti-GAD-65 limbic encephalitis (18). In this case, as steroid treatment continued throughout follow-up, albeit at tapering dosages, in parallel with PLEX, we cannot determine the effect of the individual components of the treatments.

PLEX is recommended as add-on treatment to immunosuppressives in severe and life-threatening irAEs (19), and recent American Society for Apheresis guidelines recommend PLEX as an alternative for both irAEs and para-neoplastic neurological syndromes, with GRADE 2C recommendations (20). The guidelines emphasize that removal of pathogenic autoantibodies may support PLEX in para-neoplasia while removal of the relevant ICIs, may also play a role in irAEs.

This case raises several questions, e.g., how to balance effective cancer treatment versus severe encephalitis symptoms and treatment including if durvalumab could be restarted when the SCLC progresses. Because of the severe symptoms of encephalitis, we were reluctant to reintroduce durvalumab, as reported for patients with encephalitis associated with other ICIs (21), although there is limited data on the safety of resuming ICI treatment after serious irAEs (22). It is unclear if screening for paraneoplastic antibodies before startup of ICI therapy should be recommended.

To our knowledge, this is the first case report of a patient with an n-irAE, that has been followed with serial measurements of autoantibodies from the debut of SCLC, through development and treatment of ICI-associated encephalitis. Our longitudinal data may contribute to a better understanding and awareness of the risk of using highly potent ICI treatment.

In conclusion, this case demonstrates that treatment with ICIs must be followed with vigilant monitoring for possible irAEs that can have serious or fatal consequences for the patient if left untreated.

## Data availability statement

The original contributions presented in the study are included in the article. Further inquiries can be directed to the corresponding author.

## Ethics statement

Ethical approval for reporting individual cases or case series is not required by our institution or local regulations. The patient and his wife provided their written informed consent for the publication of anonymized patient information in this article, and they received the final manuscript for comments prior to submission. Written informed consent was obtained from the participant/patient(s) for the publication of this case report.

## Author contributions

TM: Data curation, Investigation, Writing – original draft, Writing – review & editing. KS: Conceptualization, Data curation, Formal Analysis, Investigation, Methodology, Project administration, Supervision, Validation, Visualization, Writing – review & editing. AA: Data curation, Investigation, Validation, Writing – review & editing. AG: Conceptualization, Data curation, Investigation, Writing – review & editing. BG: Conceptualization, Data curation, Investigation, Writing – review & editing. KN: Conceptualization, Data curation, Investigation, Writing – review & editing. CV: Conceptualization, Data curation, Formal Analysis, Investigation, Methodology, Validation, Writing – review & editing. CL: Conceptualization, Data curation, Formal Analysis, Investigation, Methodology, Project administration, Supervision, Validation, Visualization, Writing – review & editing.
